# *Bacillus coagulans* Improves Performance by Modulating Bile Acid Metabolism to Suppress Hepatic Lipid Accumulation and Attenuating Ovarian Oxidative Stress in Late Laying Hens

**DOI:** 10.3390/ani16162466

**Published:** 2026-08-08

**Authors:** Shuaiju Guo, Zengxin Lv, Yingxuan Duan, Bangwang Peng, Junlong Niu, Yongpeng Guo, Zhixiang Wang, Wei Zhang

**Affiliations:** College of Animal Science and Technology, Henan Agricultural University, Zhengzhou 450046, Chinaguoyp@henau.edu.cn (Y.G.)

**Keywords:** *Bacillus coagulans*, production performance, lipid metabolism, ovarian function

## Abstract

In the late laying phase, laying hens often accumulate fat in their bodies due to high-fat diets, which in turn affects their production performance. *Bacillus coagulans* has good tolerance in the digestive system and is beneficial to intestinal health. The objective of this study was to evaluate the effect of *Bacillus coagulans* on production performance, lipid metabolism, and ovarian function in late-phase laying hens. The experiment involved 180 Hy-Line Grey laying hens (65 weeks of age) with similar initial egg production rates. The aged hens were randomly assigned to two treatments with six replicates of 15 hens each. A one-way experimental design was employed: CON group (basal diet) and BC group (basal diet supplemented with 3 × 10^9^ CFU/kg of *Bacillus coagulans*). The research found that adding *Bacillus coagulans* to feed significantly reduces ADFI and the feed-to-egg ratio, whilst increasing egg production (*p* < 0.05). *Bacillus coagulans* supplementation increased the abundance of Firmicutes and decreased the abundance of Actinobacteriota (*p* < 0.05), while promoting the proliferation of *Lactobacillus* and *Bacillus* (*p* < 0.05). Furthermore, *Bacillus coagulans* significantly increased the levels of the bile acids GCDCA, TUDCA, THDCA, LCA, and HDCA in the ileum (*p* < 0.05). *Bacillus coagulans* not only increased the activity of T-SOD and CAT in the ovaries, as well as the expression of the *Nrf2*, *HO-1*, and *SOD1* genes (*p* < 0.05), but also increased the expression of the estrogen receptor *ER-α*, *FSHR* and *LHR* genes (*p* < 0.05). In conclusion, dietary supplementation with *Bacillus coagulans* improved production performance by regulating hepatic lipid metabolism and enhancing ovarian function in aged laying hens.

## Simple Summary

In the late laying phase, laying hens frequently accumulate excess body fat, which can impair their reproductive health and lead to a decline in egg production. *Bacillus coagulans* is a hardy probiotic known for its strong tolerance in the digestive system and positive impacts on gut health. This study aimed to evaluate whether dietary supplementation with *Bacillus coagulans* could improve production performance, lipid metabolism, and ovarian function in 65-week-old laying hens. The results demonstrated that *Bacillus coagulans* significantly increased egg production and improved feed efficiency while decreasing daily feed intake. Additionally, supplementation positively reshaped the gut microbiota composition, elevated beneficial ileal bile acid levels, and enhanced ovarian antioxidant capacity and hormone receptor expression. In conclusion, *Bacillus coagulans* serves as an effective dietary additive to promote ovarian health and maintain optimal production performance in aged laying hens.

## 1. Introduction

In older laying hens, fatty liver syndrome is a major metabolic issue that severely impairs egg production, fertility, and hatchability [1]. The syndrome is largely triggered by high-energy feeding coupled with physical inactivity in caged environments [2]. Because the liver is the main hub for lipid metabolism, severe fat accumulation severely compromises normal liver physiological function, directly negatively impacting the entire liver–blood–ovary axis. This condition of impaired liver function not only exacerbates systemic oxidative stress but is also one of the key factors contributing to ovarian aging [3]. Concurrently, the aging process is accompanied by a continuous drop in estrogen levels [4]. Such changes disrupt the hepatic synthesis and transport of estrogen-regulated vitellogenin precursors, ultimately impairing reproductive performance during the later phases of their laying cycle. It is widely accepted that the reduced productivity observed in aged breeding birds can be attributed to ovarian aging. Studies show that ROS-driven oxidative stress contributes to ovarian aging [5]. Therefore, exploring nutritional strategies to enhance the function of the liver–blood–ovary axis in aged breeders is essential.

*Bacillus coagulans*, a spore-forming Gram-positive probiotic, combines lactic acid-producing activity with a robust coat that lets it resist heat, acid, and bile; germinate; and proliferate in the small intestine [6]. This bacterium regulates intestinal flora, enhances immunity, and boosts antioxidant function [7]. Previous studies have indicated that *Bacillus coagulans* can regulate cholesterol metabolism by binding bile acid salts. Research has found that *Bacillus coagulans* can promote the dissociation of bile salts via bile salt hydrolase, thereby regulating lipid metabolism in laying hens and reducing serum cholesterol levels [8]. Moreover, research has demonstrated that incorporating *Bacillus coagulans* into the diet markedly enhances total antioxidant capacity, anti-superoxide anion radical activity, and anti-hydroxyl radical capacity in the livers of laying hens. Additionally, it reduces the levels of ROS, malondialdehyde (MDA), and hydrogen peroxide (H_2_O_2_), thereby alleviating oxidative stress and damage within the body [9]. While prior work indicates *Bacillus coagulans* reshapes the gut microbiota, boosts antioxidant defense, and improves hepatic lipid handling, the precise mechanisms remain unclear. In this study, we hypothesized that dietary supplementation with *Bacillus coagulans* improves production performance in late-laying hens by modulating gut microbiota and bile acid composition, which subsequently suppresses hepatic lipid accumulation via the *FXR/PPARα* signaling pathway and attenuates ovarian oxidative stress via the *Nrf2* antioxidant pathway. Thus, this study aimed to investigate how dietary supplementation with *Bacillus coagulans* influences production performance, lipid metabolism, and ovarian function in late-laying hens, specifically elucidating these underlying molecular mechanisms driven by the gut–liver–ovary axis.

## 2. Materials and Methods

### 2.1. Experimental Birds and Experimental Design

*Bacillus coagulans* was procured from Henan JDZ Bio Engineering Co., Ltd., Jiaozuo, China (containing 1 × 10^10^ CFU/g of viable spores). All the hens used in this study came from the Zhenglong Agricultural and Livestock Farm in Henan, China. One hundred and eighty Hy-line Grey laying hens (65 weeks old) with similar initial egg production rates were selected for the experiment, pre-fed for two weeks (66–67 weeks), and tested for eight weeks (68–75 weeks). The hens were randomly assigned to two treatments using a computer-generated random number sequence, with six replicates of 15 hens each. A one-way experimental design was employed: CON group (basal diet) and BC group (basal diet supplemented with 3 × 10^9^ CFU/kg of *Bacillus coagulans*, equivalent to 0.3 g/kg). The basal diet used in this study was formulated based on the National Research Council (1994), as detailed in Table S1. The hens were housed in staggered three-tier cages, with each cage accommodating three laying hens. Each replicate utilized five sets of adjacent cages for a total of 15 tiers. Birds were housed under controlled conditions at 24 ± 2 °C and 52–62% relative humidity. They were exposed to a 16 h daily lighting schedule, and all hens had unrestricted access to feed and water.

### 2.2. Sample Collection

At the conclusion of the experiment, six hens with body weights around the average were randomly selected from the control group and the BC group (representing 6 independent biological replicates). After fasting for 12 h, blood samples were collected from their jugular veins. Serum was obtained via centrifugation at 3000× *g* for 10 min at 4 °C. The birds were then euthanized by cervical dislocation. Subsequently, tissue sections (0.5 cm × 0.5 cm × 0.5 cm in size) of the ileal, ovarian, and liver tissues were collected and fixed in a 4% paraformaldehyde solution for histological observation. The remaining portions of the liver and ovary (2 g), as well as the remaining ileum, were immediately snap-frozen in liquid nitrogen and stored at −80 °C or subsequent molecular and biochemical experiments.

### 2.3. Production Performance

Egg weights and quantities were recorded daily, whereas feed consumption was assessed weekly for each replicate to determine average daily feed intake (ADFI), average egg weight, egg production rate, and feed-to-egg ratio. These metrics were calculated for the periods of days 1–28, 28–56, and 1–56 of the experiment. The calculations were performed based on the following established formulas [10,11]:

Average egg weight (g) = Total egg weight/Total number of eggs(1)

Egg production rate (%) = (Total number of eggs/(Number of hens × Days)) × 100(2)

ADFI (g/bird/day) = Total feed consumption/(Number of hens × Days)(3)

Egg mass (g/bird/day) = (Egg production rate × Average egg weight)/100(4)

Feed-to-egg ratio = ADFI/Egg mass(5)

### 2.4. Serum Hormone Levels and Ovarian Antioxidant Status

Serum total cholesterol (TC), triglycerides (TG), high-density lipoprotein (HDL), low-density lipoprotein (LDL), aspartate aminotransferase (AST), alanine aminotransferase (ALT), follicle-stimulating hormone (FSH), luteinizing hormone (LH), estradiol (E_2_), and progesterone (P_4_) were measured using a fully automated biochemical analyzer (Shenzhen Mindray Bio-Medical Electronics Co., Ltd., Shenzhen, China BS-360S). Approximately 0.2 g of preserved ovarian tissue was weighed and homogenized in 2 mL of ice-cold PBS. Following centrifugation at 12,000× *g* for 10 min at 4 °C, the supernatants were collected for oxidative status analysis. The levels of total antioxidant capacity (T-AOC), glutathione peroxidase (GPX), catalase (CAT), and malondialdehyde (MDA) in ovarian tissue were quantified using kits from Nanjing Jiancheng Bioengineering Institute (Nanjing, China) (catalog numbers A015-2-1, H545-1-1, A007-1-1, A003-3-1).

### 2.5. Histology of Liver and Ovary

Liver and ovarian samples from six birds per group were fixed in 4% paraformaldehyde and paraffin-embedded. Paraffin blocks were cut into 5 µm sections and hematoxylin–eosin-stained for histomorphometry [5]. For ovarian tissue, six fields of view were randomly selected from each section for imaging, and the number of primordial follicles was recorded. For liver tissue, six fields of view were randomly selected from each section for microscopic examination to assess the degree of fatty degeneration. Liver slices were processed as for HE staining, rinsed 3 times for 5 min in PBS, and then briefly dipped in 60% isopropanol to enhance Oil Red O staining [12]. The slices were then incubated in Oil Red O/isopropanol solution for 15 min in the dark. After staining, the slides were briefly rinsed in isopropanol to adjust the color intensity. The nuclei were rinsed under running water and stained with hematoxylin staining solution. The sections were washed again under running water and treated with 1% hydrochloric acid in alcohol for 1–2 s. Finally, the sections were thoroughly rinsed with running water, drained, sealed, stored under refrigeration, observed with a light microscope (Olympus, Tokyo, Japan) equipped with a DP73 digital camera, and photographed using cellSens Standard imaging software version 4.1.

### 2.6. TUNEL Assay of Ovarian Tissue

The paraffin sections were prepared according to the procedure outlined in Section 2.5. TUNEL staining was performed with kit A113 (Vazyme Biotech, Nanjing, China) following the supplier protocol. The brightness (green) of apoptotic cells was quantified using ImageJ software (version 1.53, NIH, Bethesda, MD, USA). To prevent subjective bias, all histological samples were coded, and the investigators performing the microscopic observations, imaging, and quantitative analyses were strictly blinded to the treatment allocations. Unblinding was performed only after all data acquisition was complete.

### 2.7. Hepatic, Ileal, and Ovarian Gene Expression Levels in Laying Hens

Real-time quantitative PCR (qPCR) analysis of hepatic, ileal, and ovarian samples was performed as previously described [13]. Total RNA was extracted from liver, ileum, and ovary samples of six birds per group (TaKaRa, Dalian, China), and its integrity, concentration, and purity were verified with a NanoDrop spectrophotometer (Thermo Fisher Scientific, Waltham, MA, USA) [14]. cDNA was synthesized using a cDNA synthesis kit (TaKaRa, Dalian, China). qRT-PCR was run on an Agilent Mx3000P system (Agilent Technologies, Santa Clara, CA, USA). The PCR cycling conditions were as follows: initial denaturation at 95 °C for 3 min, followed by 40 cycles of denaturation at 95 °C for 5 s, and annealing/extension at 60 °C for 30 s. In this experiment, all samples were replicated three times, and the relative abundance of each transcript was normalized using β-actin. Relative mRNA expression of target genes was calculated using the 2^−ΔΔCt^ method. Table S2 provides the primer sequences for hepatic lipid metabolism-related genes (*FXR*, *ACC*, *FAS*, *SREBP1*, *LXRα*, *ACOX1*, *CYP7A1*, *PPARα*, *PPARγ*, *APOB*, *APOV1*, and *VTG2*) and ovarian estrogen receptor (ER) and antioxidant pathway genes (*ER-α*, *ER-β*, *FSHR*, *LHR*, *Nrf2*, *HO-1*, *NQO1*, *GPX1*, and *SOD1*) utilized in qPCR.

### 2.8. Structure of Intestinal Flora

Ileal content samples were analyzed for microbiota as previously described [15]. Ileal-content DNA from 5 birds per group was isolated with a Tiangen kit (Beijing, China) per the supplier protocol. DNA concentration was measured with a NanoDrop, and integrity was checked by agarose gel electrophoresis. The samples were then diluted to a specific concentration using sterile water. The diluted genomic DNA served as a template for amplification using universal primers 338F (5′-ACTCCTACGGGAGGCAGGA-3′) and 806R (5′-GGACTACHVGGGTWTCTTAT-3′), targeting the V3–V4 region of the 16S rDNA gene. The PCR products were sequenced on an Illumina HiSeq 2500 PE250 platform using sequencing by synthesis technology (Shanghai Paisano Biotechnology Co., Ltd., Shanghai, China). Following sequencing, the raw reads were processed using the Qiime2 pipeline (version 2022.2). Quality control, denoising, and chimera removal were strictly performed using the DADA2 plugin to generate Amplicon Sequence Variants (ASVs). To normalize uneven sequencing depth for diversity analyses, the ASV table was rarefied to 20,000 sequences per sample. Finally, taxonomic classification of the ASVs was conducted against the Silva 16S rRNA gene database (version 138.2) utilizing a Naive Bayes classifier within Qiime2 (version 2022.2, Northern Arizona University, Flagstaff, AZ, USA).

### 2.9. Intestinal Bile Acid Composition

About 30 mg of ileal digesta was collected from each of the five birds per group that were previously euthanized. No additional birds were euthanized for this purpose. Ileal digesta samples (20 mg) were extracted with 495 µL of methanol. Following this, 5 µL of an internal standard mixed solution (10 µg/mL; standards purchased from CNW and IsoReag, Shanghai, China) was added to the extract as the internal standard for accurate quantification. The mixture was vortexed for 30 s, milled with steel beads for 4 min, and then sonicated for 5 min in an ice bath. Grinding and ultrasonic treatment were repeated three times, after which the sample was placed at −20 °C for one hour before centrifugation. The supernatant was then collected in injection vials for ultra-high performance liquid chromatography–mass spectrometry (UHPLC-MS/MS) analysis using a QTRAP 6500+ LC-MS/MS System (AB Sciex, Framingham, MA, USA). Using a Waters ACQUITY UPLC HSS T3 C18 column (1.8 µm, 100 mm × 2.1 mm i.d.), mobile phase A was ultrapure water (containing 0.01% acetic acid and 5 mmol/L ammonium acetate), and mobile phase B was acetonitrile (containing 0.01% acetic acid). A linear gradient was employed during elution: 0 min A/B = 95:5 (*v*/*v*), 1 min A/B = 60:40 (*v*/*v*), 7 min A/B = 50:50 (*v*/*v*), 12 min A/B = 25:75 (*v*/*v*), 14 min A/B = 5:95 (*v*/*v*), and 16.0 min A/B = 95:5 (*v*/*v*). The flow rate was 0.35 mL/min, the column temperature was 40 °C, and the injection volume was 3 µL. For precise targeted quantification, a total of 82 bile acids were quantified utilizing the Metware Database (MWDB) constructed from standard samples. Calibration curves were generated using standard solutions prepared at 15 concentration levels ranging from 0.1 ng/mL to 4000 ng/mL. Linear equations were derived by plotting the external standard concentration against the peak area ratio of the standard to the internal standard. To monitor instrument stability, quality control (QC) samples, prepared from a mixed solution, were injected every 10 samples throughout the analytical run. Finally, data normalization and absolute quantification were achieved by substituting the integral peak area ratio of the sample into the corresponding standard curve linear equation to calculate the concentration, which was then normalized by the extraction volume and the initial sample mass.

### 2.10. Statistical Analysis

Statistical analyses were conducted using SPSS 26.0 software. Prior to conducting the comparative analysis, the normality of the data distribution and the homogeneity of variances were verified (*p* > 0.05). Having confirmed these parametric assumptions, the statistical significance between the two groups was assessed using an independent samples *t*-test. A *p* < 0.05 was considered statistically significant, and results were expressed as mean ± SEM. Histograms were generated using GraphPad Prism version 8.

In the analysis of the gut microbiota, the raw sequencing read data were quality-filtered using fastp (version 0.2). Subsequently, the optimized sequences were processed using the DADA2 plugin within the QIIME2 pipeline to identify amplicon sequence variants (ASVs). Taxonomic assignment of the ASVs was performed against the Silva 16S rRNA gene database (v138.2) using a Naive Bayes classifier. Microbiome data analyses were carried out on the Majorbio Cloud Platform. Alpha diversity indices, including ACE, Chao1, Shannon, and Simpson, were calculated using mothur software (version 1.30.2). Beta diversity was evaluated by Principal Coordinate Analysis (PCoA) and Non-metric Multidimensional Scaling (NMDS) based on weighted UniFrac distances. Linear discriminant analysis Effect Size (LEfSe) was performed to identify significantly differential bacterial taxa between groups, with a logarithmic LDA score threshold set to >2.0.

For the targeted bile acid metabolomics, the LC-MS/MS raw data were processed using Analyst 1.6.3 software (AB Sciex) for data acquisition and MultiQuant 3.0.3 software (AB Sciex) for peak extraction, integration, and quantitative calibration. Prior to multivariate analysis, the data underwent unit variance scaling (UV) and mean centering. Unsupervised Principal Component Analysis (PCA) was performed using the prcomp function in R (Version 3.5.1) to visualize overall metabolic profiling. Supervised Orthogonal Partial Least Squares–Discriminant Analysis (OPLS-DA) was executed using the R package MetaboAnalystR to maximize group separation and identify key variables. Differentially expressed metabolites were selected based on the criteria of VIP > 1, *p* < 0.05, and FC ≥ 2 or FC ≤ 0.5.

## 3. Results

### 3.1. Effect of Bacillus coagulans on Production Performance of Aged Laying Hens

Compared with the CON group (Table 1), the BC group showed a significant decrease in ADFI across all three periods (*p* < 0.05), a significant increase in egg production from days 1 to 28 and from days 1 to 56 (*p* < 0.05) but no significant change from days 28 to 56 (*p* > 0.05), and a significant decrease in the feed-to-egg ratio from days 28 to 56 and from days 1 to 56 (*p* < 0.05). Specifically, over the entire 56-day experimental period (1–56D), dietary supplementation with *Bacillus coagulans* resulted in a 2.52% relative improvement in egg production and a 5.80% enhancement in feed conversion efficiency compared to the CON group.

### 3.2. Effect of Bacillus coagulans on Serum Lipid Parameters and Hepatic Health

**Figure 1:**
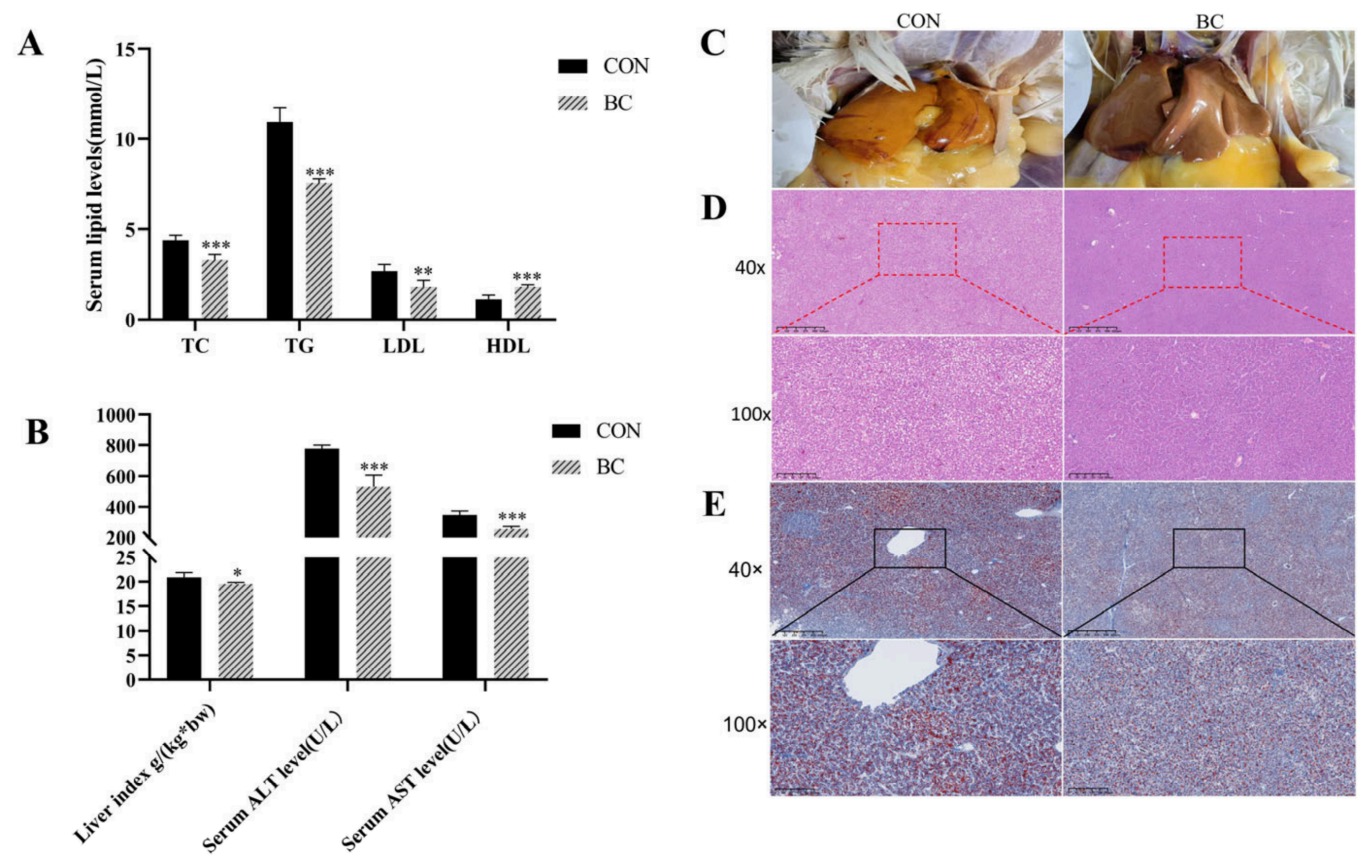
Effect of *Bacillus coagulans* on serum lipid-metabolizing lipid profiles and liver injury. (**A**) Serum lipid levels. (**B**) Liver index and serum liver damage indicators. (**C**) Field deconstruction image of the liver. (**D**) HE-stained section of the liver (100×: scale bar = 200 μm; 40×: scale bar = 625 μm). (**E**) Oil red O-stained section of the liver (100×: scale bar = 200 μm; 40×: scale bar = 625 μm). All data are expressed as mean ± SEM, n = 6. * *p* < 0.05, ** *p* < 0.01, *** *p* < 0.001. CON: basal diet; BC: basal diet + 3 × 10^9^ CFU/kg *Bacillus coagulans*.

As shown in Figure 1, compared to the control group, dietary supplementation with *Bacillus coagulans* significantly decreased the serum levels of TC, TG, and LDL by absolute values of 1.11 mmol/L (from 4.41 to 3.30), 3.39 mmol/L (from 10.96 to 7.57), and 0.89 mmol/L (from 2.69 to 1.80), respectively, whereas HDL levels significantly increased (*p* < 0.05) (Figure 1A). Furthermore, the BC group showed a significantly lower liver index, along with absolute reductions in serum ALT and AST activities by 247.12 U/L (from 777.20 to 530.08) and 91.26 U/L (from 345.75 to 254.49), respectively (*p* < 0.05) (Figure 1B). The liver dissection image shows that control group livers were pale yellow with hemorrhage, whereas *Bacillus coagulans* group livers exhibited a normal reddish-brown color (Figure 1C). HE staining revealed significant inflammatory cell infiltration in the control group, characterized by numerous lipid vesicles and severe vacuolar degeneration (Figure 1D). Oil Red O staining revealed widespread steatosis and lipid accumulation in control livers, an effect markedly attenuated by *Bacillus coagulans* supplementation (Figure 1E).

### 3.3. Effect of Bacillus coagulans on Genes Related to Hepatic Lipid Metabolism

**Figure 2:**
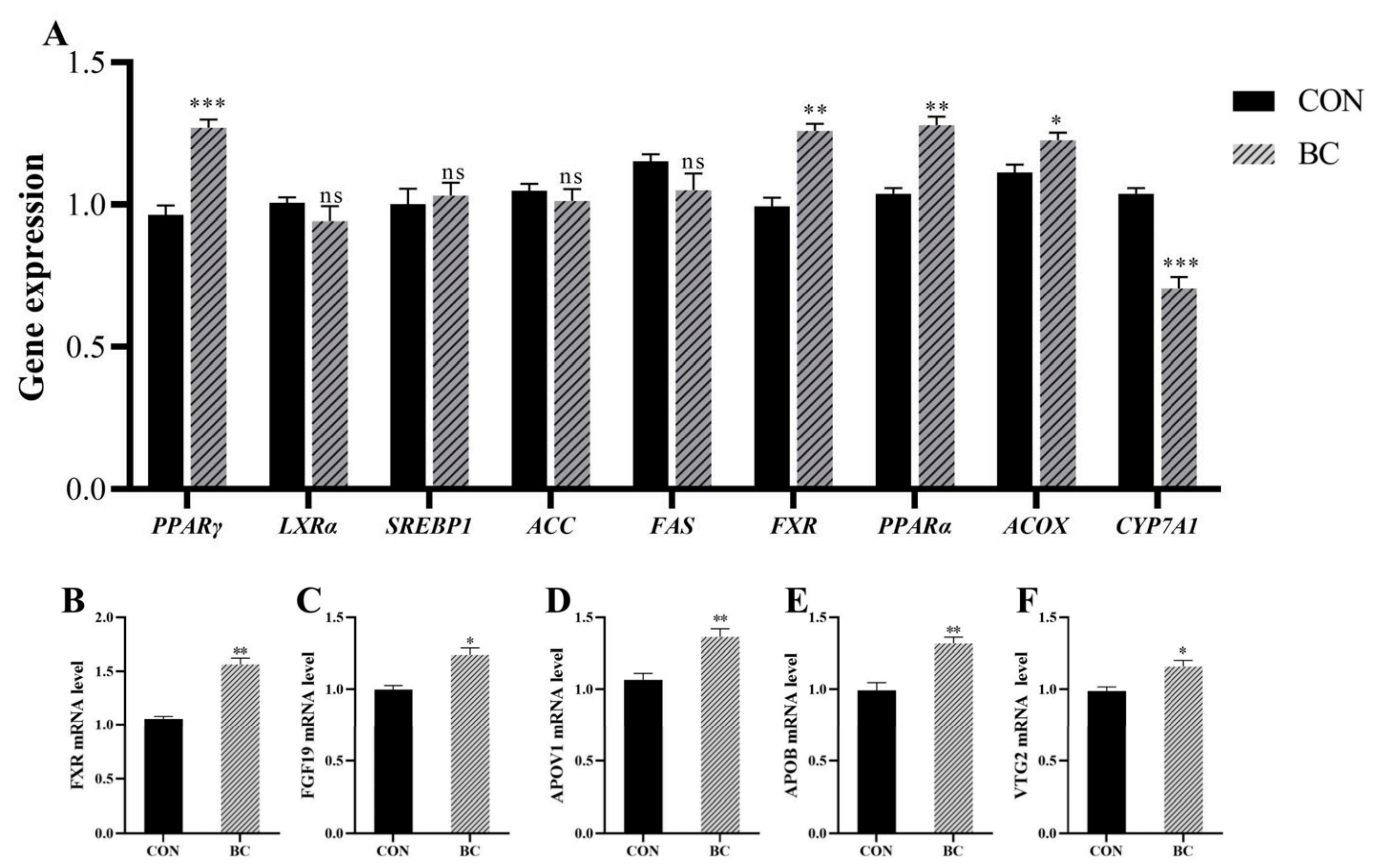
Effect of *Bacillus coagulans* on the expression of lipid metabolism-related genes in the liver and ileum. (**A**) Expression of genes involved in lipid metabolism. (**B**,**C**) FXR receptor gene expression. (**D**–**F**) Apolipoprotein gene expression. All data are expressed as mean ± SEM, n = 6. ns *p* > 0.05, no significant difference; * *p* < 0.05, ** *p* < 0.01, *** *p* < 0.001. CON: (basal diet); BC: (basal diet + 3 × 10^9^ CFU/kg *Bacillus coagulans*).

Figure 2 shows hepatic *FXR*, *PPARα*, and *ACOX1*—key genes driving fatty-acid oxidation—were all significantly upregulated by 1.27-fold, 1.23-fold, and 1.10-fold, respectively, in BC birds versus controls (Figure 2A). Additionally, the expressions of *FXR* and *FGF19* genes were also significantly elevated in the ileum by 1.48-fold and 1.24-fold, respectively (*p* < 0.05) (Figure 2B,C). Conversely, the rate-limiting enzyme for bile acid synthesis, *CYP7A1*, significantly decreased to 0.68-fold of the control, while the expression of apolipoprotein genes *APOV1*, *APOB*, and *VTG2* significantly increased by 1.28-fold, 1.33-fold, and 1.17-fold, respectively (*p* < 0.05) (Figure 2D–F).

### 3.4. Effect of Bacillus coagulans on Intestinal Microbial Diversity in Aged Laying Hens

**Figure 3:**
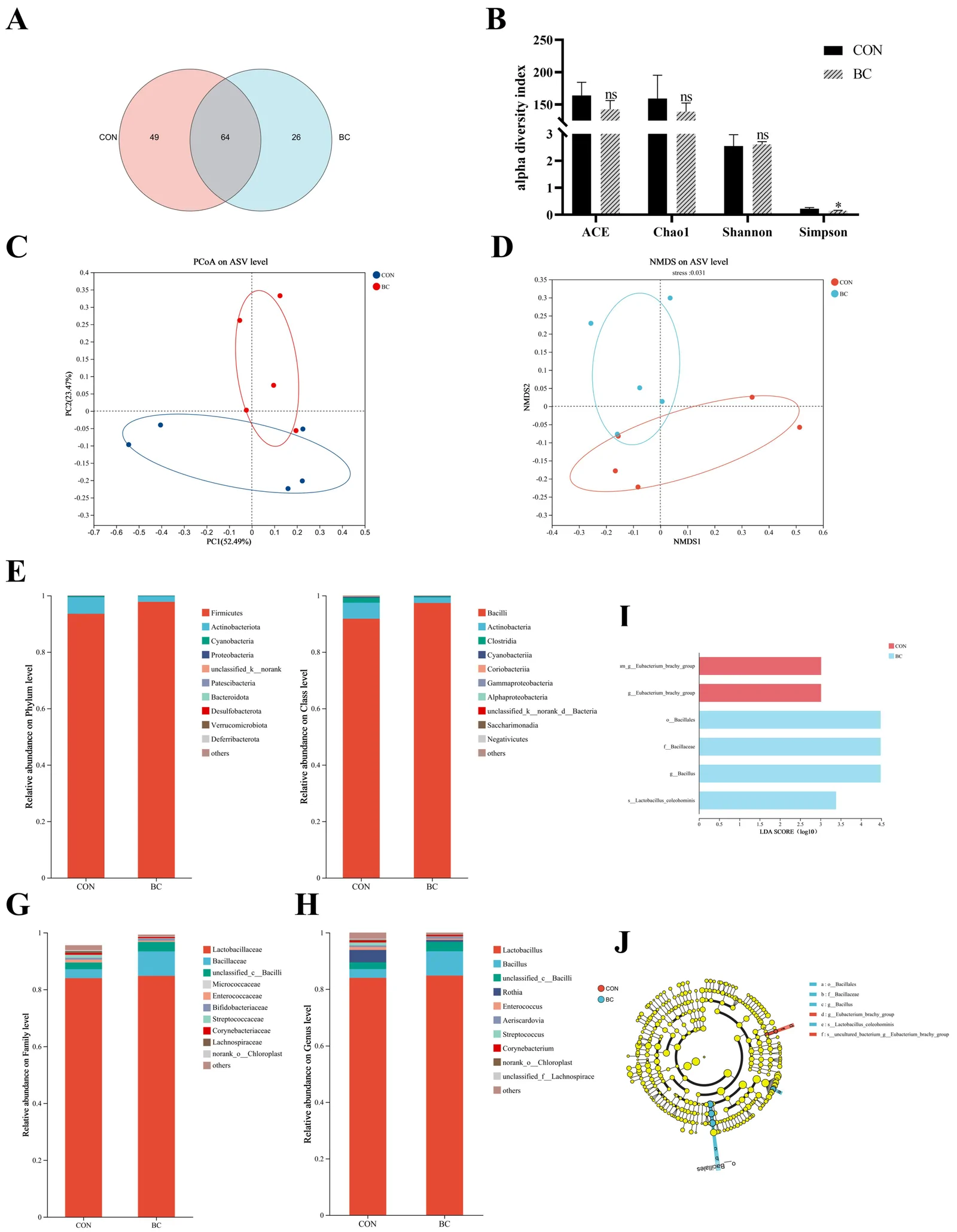
Effect of *Bacillus coagulans* on microbial diversity. (**A**) Venn diagram showing unique and shared ASVs among the four treatment groups. (**B**) Comparison of alpha diversity indices, including ACE index, Chao1 index, Shannon index, and Simpson index. (**C**,**D**) Weighted UniFrac distance matrix analysis visualized by PCoA and NMDS, with each point representing a sample and colors indicating different groups. (**E**–**H**) Composition of microbial communities at the phylum, order, family, and genus levels. Color bar graphs showing the relative abundance of microbial taxa. (**I**,**J**) LEfSe analysis identifying taxa of differential abundance in the ileal chyme microbial community, displaying only taxa with LDA scores greater than 2. All data are expressed as mean ± SEM, n = 5. ns *p* > 0.05, no significant difference; * *p* < 0.05. CON: basal diet; BC: basal diet + 3 × 10^9^ CFU/kg *Bacillus coagulans*.

The structural analysis of ileal flora in the control and *Bacillus coagulans* groups revealed that there were 64 shared ASVs between the two groups (Figure 3A). The alpha diversity index indicates a significant decline in the Simpson’s index for the BC group (*p* < 0.05) (Figure 3B). The beta diversity Principal Coordinate Analysis demonstrated that *Bacillus coagulans* supplementation significantly altered the intestinal microbial community composition, resulting in a distinct clustering separation between the two groups (Figure 3C,D). At the phylum level, the microbial community of the laying hen ileum was predominantly composed of Firmicutes, followed by Actinobacteria. At the family level, the ileal microbial community was primarily dominated by Lactobacillaceae, with Firmicutes following closely. At the genus level, the genus *Lactobacillus* dominated the community, followed by the *genus* Bacillus within the family Bacillaceae (Figure 3E–H).

Table S3 shows that *Bacillus coagulans* significantly raised ileal Firmicutes and Bacilli levels while significantly lowering Actinobacteriota and Actinobacteria (*p* < 0.05). Furthermore, *Bacillus coagulans* significantly increased the relative abundance of Bacillales and Bifidobacteriales, concurrently decreasing the relative abundance of Micrococcales and Lachnospirales (*p* < 0.05). The supplementation of *Bacillus coagulans* also resulted in a significant increase in the relative abundance of Lactobacillaceae, Bacillaceae, Micrococcaceae, Bifidobacteriaceae, as well as Lactobacillus and Bacillus, while significantly decreasing the relative abundance of Streptococcaceae, Rothia, and Streptococcus (*p* < 0.05). The LEfSe analysis further indicated that the ileum of one aged laying hen in the experimental group was significantly enriched in *Bacillus* and *Lactobacillus*, likely attributable to the increased presence of *Bacillus coagulans* in the ileum (Figure 3I,J).

### 3.5. Effect of Bacillus coagulans on Intestinal Bile Acid Composition in Aged Laying Hens

**Figure 4:**
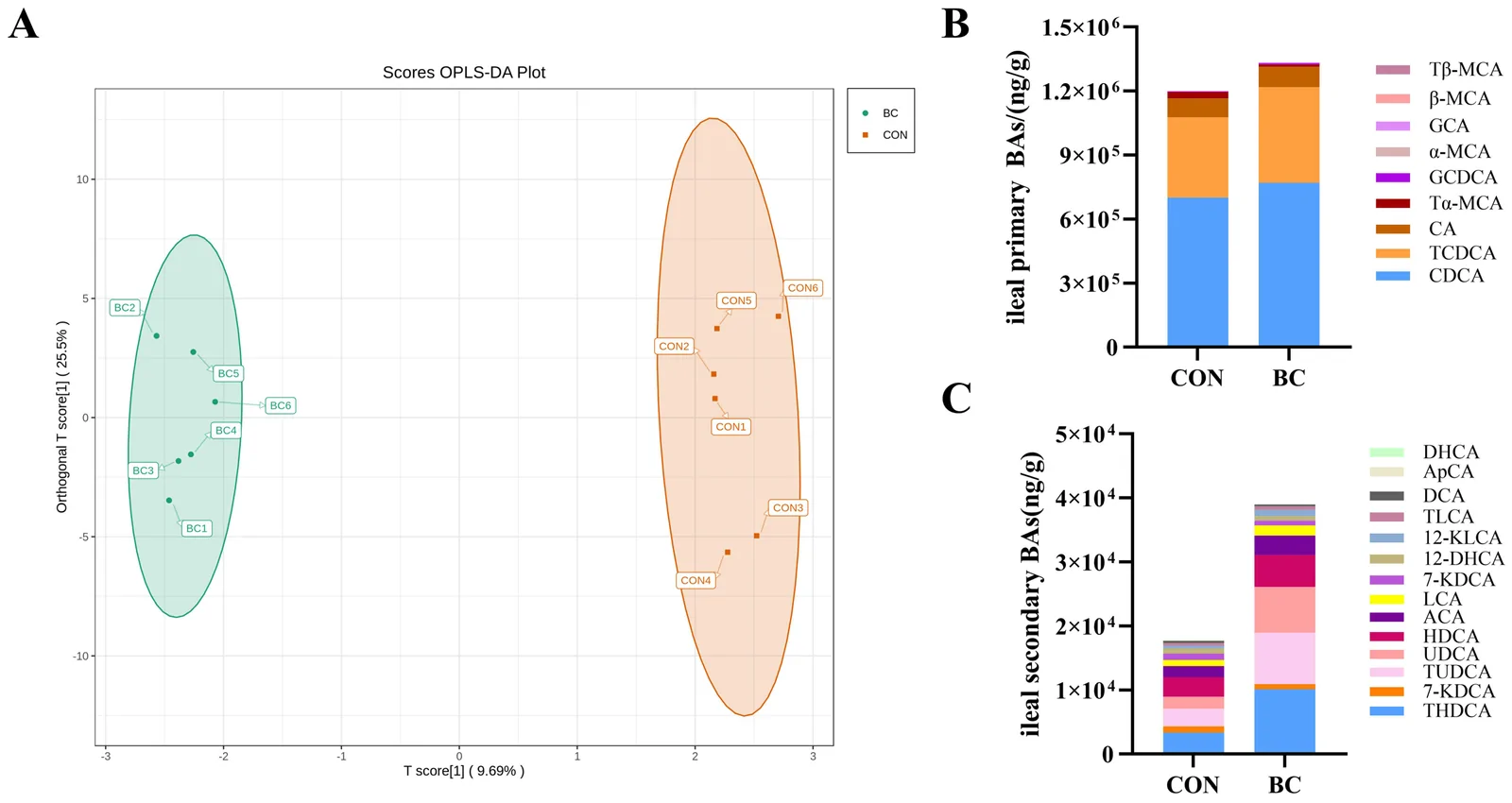
Effect of *Bacillus coagulans* on ileal bile acid composition in aged laying hens. (**A**) Orthogonal partial least squares–discriminant analysis (OPLS-DA) of the bile acid composition of ileal chyme. (**B**,**C**) Composition of primary and secondary bile acids in the ileal contents. CON: basal diet; BC: basal diet + 3 × 10^9^ CFU/kg *Bacillus coagulans*.

OPLS-DA highlighted distinct ileal bile acid signatures between control and *Bacillus coagulans*-fed late laying hens (Figure 4A). The analysis indicated that primary bile acids in the ileum predominantly comprise CDCA, TCDCA, and CA, whereas secondary bile acids were primarily represented by THDCA, TUDCA, and HDCA (Figure 4B,C). As illustrated in Table S4, the inclusion of *Bacillus coagulans* significantly altered the absolute concentrations of specific bile acids. Compared to the control group, the absolute concentrations of ileal GCDCA, TUDCA, THDCA, LCA, and HDCA were significantly increased, whereas the concentration of tauro-α-muricholic acid (Tα-MCA) was significantly decreased (*p* < 0.05). Notably, when evaluating the magnitude of these shifts among the significantly regulated metabolites, GCDCA exhibited the largest fold increase (3.45-fold), while Tα-MCA showed the most substantial reduction (decreasing to 0.37-fold of the control level).

### 3.6. Analysis of the Correlation Between Intestinal Flora and Bile Acids

**Figure 5:**
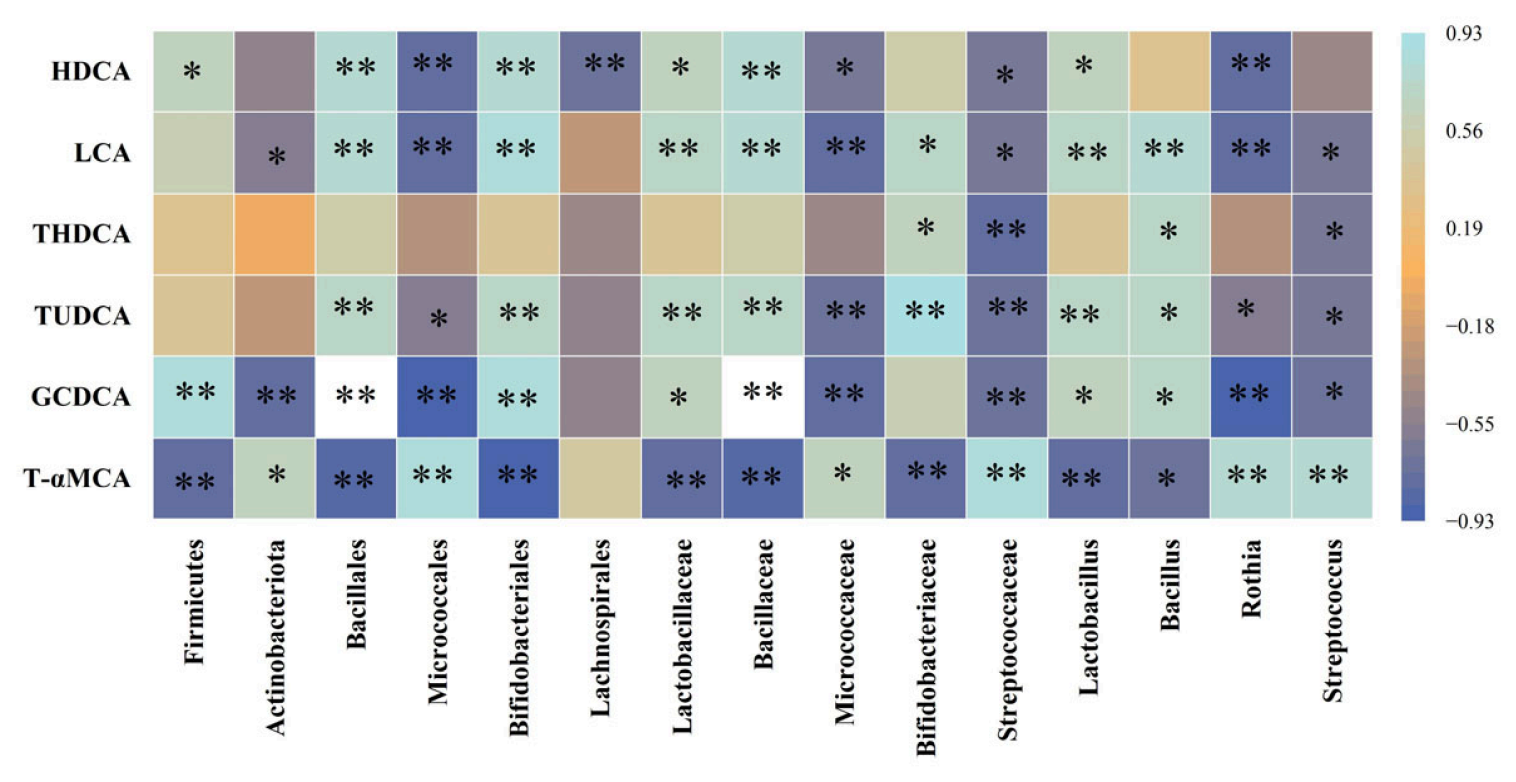
Spearman correlation analysis between differential bacteria and bile acids. * *p* < 0.05, ** *p* < 0.01.

Spearman’s rank test was used to assess links between ileal microbiota and bile acid levels (Figure 5). The results at the phylum and genus levels indicated that T-αMCA exhibited a positive correlation with Actinobacteriota (*R* = 0.54, *p* < 0.05), Rothia (*R* = 0.78, *p* < 0.01), and Streptococcus (*R* = 0.81, *p* < 0.01), and a negative correlation with Firmicutes (*R* = −0.83, *p* < 0.01), Lactobacillus (*R* = −0.90, *p* < 0.01), and Bacillus (*R* = −0.73, *p* < 0.05). GCDCA levels rose alongside Firmicutes (*R* = 0.86, *p* < 0.01), Lactobacillus (*R* = 0.71, *p* < 0.05) and Bacillus (*R* = 0.75, *p* < 0.05) but declined with Actinobacteriota (*R* = −0.89, *p* < 0.01), Rothia (*R* = −0.93, *p* < 0.01) and Streptococcus (*R* = −0.74, *p* < 0.05). TUDCA presented a positive correlation with Lactobacillus (*R* = 0.87, *p* < 0.01) and Bacillus (*R* = 0.73, *p* < 0.05), and a negative correlation with Rothia (*R* = −0.53, *p* < 0.05) and Streptococcus (*R* = −0.81, *p* < 0.01). LCA demonstrated a positive correlation with Lactobacillus (*R* = 0.87, *p* < 0.01) and Bacillus (*R* = 0.90, *p* < 0.01), and a negative correlation with Rothia (*R* = −0.88, *p* < 0.01) and Streptococcus (*R* = −0.72, *p* < 0.05). THDCA was positively correlated with Bacillus (*R* = 0.74, *p* < 0.05) and negatively correlated with Streptococcus (*R* = −0.73, *p* < 0.05). HDCA levels rose with Firmicutes (*R* = 0.69, *p* < 0.05) and Lactobacillus (*R* = 0.70, *p* < 0.05) yet fell with Rothia (*R* = −0.88, *p* < 0.01).

### 3.7. Effect of Bacillus coagulans on Ovarian Function

**Figure 6:**
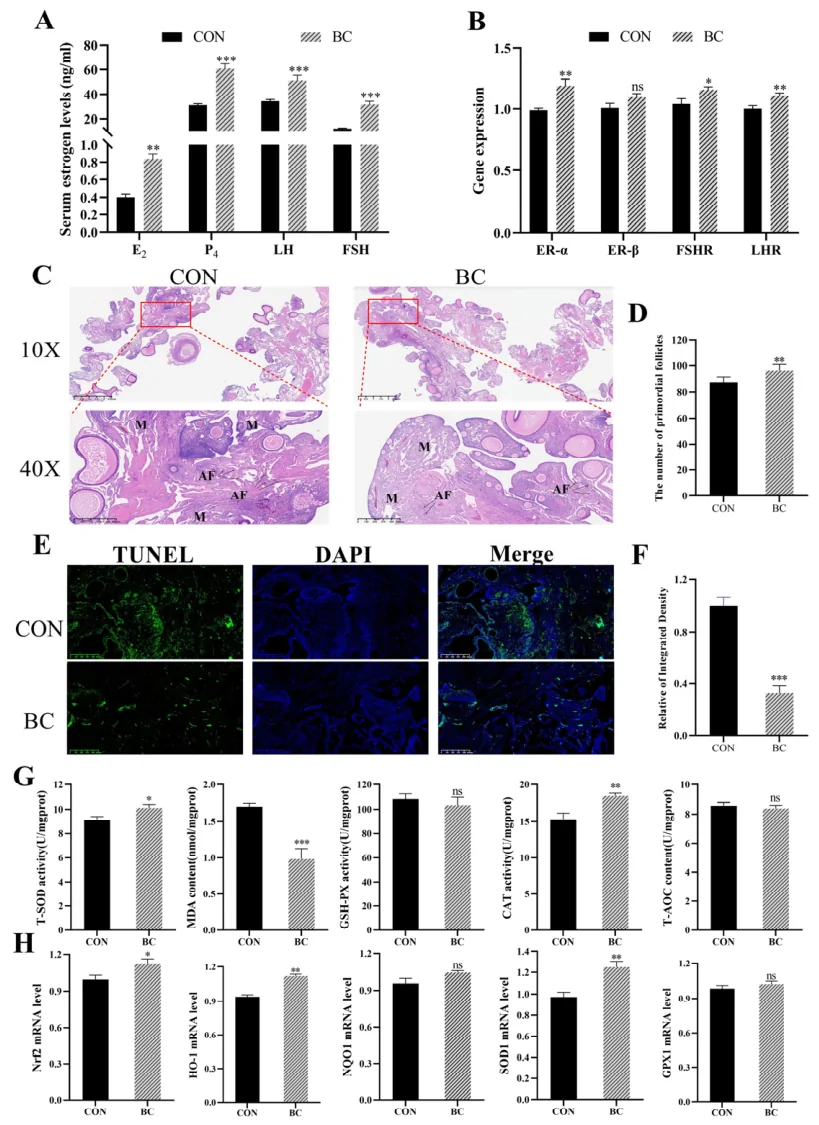
Effect of *Bacillus coagulans* on ovarian function. (**A**) Serum estrogen levels. (**B**) Relative expression levels of estrogen receptor genes. (**C**) Ovarian histomorphology (scale bar = 625 μm). AF, atretic follicles; M, medullary structures. (**D**) Statistical analysis of the number of primordial follicles in the ovary. (**E**) TUNEL assay of ovary (scale bar = 625 μm). (**F**) Statistical analysis of apoptotic index in ovary. (**G**,**H**) Ovarian antioxidant capacity. All data are expressed as mean ± SEM, n = 6. ns *p* > 0.05, no significant difference; * *p* < 0.05, ** *p* < 0.01, *** *p* < 0.001. CON: basal diet; BC: basal diet + 3 × 10^9^ CFU/kg *Bacillus coagulans*.

As illustrated in Figure 6, the *Bacillus coagulans* group exhibited a marked elevation in serum E_2_, P_4_, LH, and FSH levels relative to the control group (*p* < 0.05) (Figure 6A). Compared to the CON group, the BC group showed a significant upregulation in ovarian *ER-α*, *FSHR*, and *LHR* gene expression (*p* < 0.05) (Figure 6B). Figure 6C shows that ovarian architecture remained intact across all groups, with primordial follicles present in varying quantities. However, the medullary structure appeared more compact in the *Bacillus coagulans* group (M), in contrast to the CON group, which exhibited a higher incidence of atretic follicles (AF). In addition, we conducted a quantitative analysis of the number of primordial follicles in the ovaries and found that supplementation with *Bacillus coagulans* significantly increased the number of primordial follicles from 87.28 ± 1.67 in the CON group to 96.36 ± 1.85 in the BC group, thereby slowing down the atresia and loss of primordial follicles (*p* < 0.05) (Figure 6D). A TUNEL assay was conducted to evaluate the effect of *Bacillus coagulans* on the ovarian apoptosis index. The relative integrated density of apoptotic cells decreased significantly from 1.00 ± 0.07 in the CON group to 0.33 ± 0.06 in the BC group, revealing that ovarian apoptosis was significantly alleviated (*p* < 0.05) (Figure 6E,F). The results of the ovarian antioxidant marker tests show that *Bacillus coagulans* significantly elevated T-SOD and CAT activities while significantly lowering ovarian MDA content (*p* < 0.05) (Figure 6G). Additionally, the expression of the *Nrf2*, *HO1*, and *SOD1* genes in the *Nrf2* signaling pathway was significantly upregulated (*p* < 0.05) (Figure 6H).

## 4. Discussion

During the late egg-laying phase, laying hens frequently experience a marked decline in performance due to intensive farming practices and high-fat diets [16]. Emerging studies indicate that as laying hens age, they often exhibit diminished efficiency in liver lipid handling and ovarian performance [17]. Such changes typically coincide with marked reductions in antioxidant reserves, reproductive hormone output, and follicular maturation. Ultimately, this sequence of events results in reduced production of yolk precursors, adversely affecting overall egg yield [18,19]. Moreover, dietary supplementation with Bacillus subtilis has been shown to significantly enhance egg production rates, eggshell quality, and blood biochemical indices in laying hens [20]. In the current study, dietary supplementation with *Bacillus coagulans* significantly reduced ADFI and the feed-to-egg ratio but increased egg production. The enhancement in egg-laying performance may be attributed to improved liver and ovarian function. Serum levels of cholesterol, TG, LDL, and HDL are commonly utilized indicators of lipid metabolism efficiency [21]. Research has shown that administering *Bacillus coagulans* orally to broiler chickens for 42 consecutive days results in a significant reduction in serum LDL and TG levels, and a significant increase in TP and ALB levels [22]. In this study, the addition of *Bacillus coagulans* significantly reduced serum TC, TG, and LDL levels, indicating that *Bacillus coagulans* plays a role in regulating lipid metabolism. In poultry, the liver is the main hub for lipid metabolism; excessive hepatic fat can trigger cell damage and steatosis [23,24]. AST and ALT are widely recognized indicators of liver function, used to assess hepatic health. AST participates in amino acid metabolism, catalyzing the enzymatic transfer reaction between aspartate and oxaloacetate, whereas ALT facilitates the transfer reaction between alanine and α-ketoglutarate [25]. Furthermore, fatty liver is frequently accompanied by marked inflammatory cell infiltration within the hepatic tissue. Probiotic supplementation has been found to significantly reduce AST and ALT levels, suggesting potential improvements in liver function [26]. Additionally, dietary supplementation with Lactobacillus and Bacillus subtilis has been shown to enhance liver function and decrease serum levels of ALT and AST in laying hens [27]. In the present study, the BC group exhibited reduced inflammatory cell infiltration in the liver of laying hens during the late laying stage, with decreased liver index, AST, and ALT levels, corroborating findings from previous research.

Hepatic fat content is primarily influenced by the balance between fatty acid synthesis and oxidation. *PPARγ* serves as a major regulator of adipocyte differentiation and adipogenesis, facilitating adipocyte formation and lipid storage [28]. *ACC* and *FAS* are pivotal enzymes for de novo fatty acid synthesis and dictate adipogenic potential [29]. *LXRα*, once activated, boosts *SREBP1* expression, which in turn switches on *ACC* and *FAS* transcription to accelerate lipogenesis. Here, *PPARγ* expression rose markedly, yet the downstream lipogenic genes *LXRα*, *SREBP1*, *ACC*, and *FAS* remained unchanged. Therefore, these findings suggest that *Bacillus coagulans* does not facilitate lipid synthesis through the *PPARγ/LXRα/SREBP1* pathway. Beyond adipogenesis, *PPARγ* is also a potent anti-inflammatory factor. Its upregulation likely serves a hepatoprotective role to resolve liver injury, which perfectly aligns with the markedly reduced inflammatory cell infiltration observed in our histological analysis. Consequently, with lipogenesis unactivated and the *FXR/PPARα/ACOX* pathway robustly driving fatty acid β-oxidation, the net metabolic balance shifts toward lipid catabolism, thereby reducing overall hepatic fat accumulation. *ACOX* comprises a class of enzymes that play a critical role in fatty acid β-oxidation, predominantly located in the peroxisomes of cells [30]. *ACOX* contains the response element of PPAR, and its catalytic fatty acid oxidation relies on the action of *PPARα*. *FXR*, a receptor for bile acids, significantly regulates lipid metabolism within the organism [31]. Studies show that reduced *FXR* expression parallels hepatic steatosis, and *FXR* silencing alone provokes lipid buildup in liver and plasma [32]. Conversely, the activation of *FXR* enhances fatty acid oxidation through the upregulation of PPARα and reduces hepatic fat deposition [33]. In the present experiment, the upregulation of *ACOX*, *PPARα*, and *FXR* expression suggests that *Bacillus coagulans* may enhance β-oxidation of fatty acids in the liver of laying hens via the *FXR/PPARα/ACOX* pathway. This effect may be more pronounced than that of fatty acid synthesis, contributing to a reduction in serum TC and TG levels.

Gut microbial makeup strongly influences host lipid metabolism. *Bacillus coagulans* raised Firmicutes and lowered Actinobacteria, changes that may curtail fat deposition. Previous studies have shown that bacteria of the Lactobacillaceae family (such as Lactobacillus), which were significantly enriched in the BC group, are associated with lipid metabolism. In the present study, this enrichment was significantly correlated with the observed changes in lipid synthesis markers, suggesting that they may exert a synergistic effect [34]. Bacillaceae have also demonstrated significant regulatory roles in lipid metabolism, capable of modulating lipid synthesis and catabolism, improving blood lipid levels, and enhancing antioxidant capacity [35]. Dietary Bacillus subtilis markedly lowered lipid droplet size and hepatic fat in grass carp, as Oil Red O staining showed, while it suppressed *ACC* expression and lifted LPL and ATGL levels [36]. Bacteria from the families Micrococcaceae and Bifidobacteria may influence lipid absorption and cholesterol metabolism by regulating bile acid metabolism. In this experiment, the increase in intestinal Lactobacillaceae, Bacillaceae, and Bifidobacteriaceae suggests that *Bacillus coagulans* can alleviate the body’s oxidative stress state, reduce susceptibility to lipid deposition, and contribute to the reduction in fat accumulation. Furthermore, Lactobacillus have been shown to significantly reduce adiposity, increase fecal fat excretion, and improve obesity-related muscle health and function in obese mice [37]. Additionally, Lactobacillus spp. can positively modulate the composition of the intestinal flora, demonstrating potential as an anti-obesity and anti-muscle loss agent. Lactobacillus likewise mitigates atherosclerosis by activating the *Nrf2/HO-1* axis, thereby dampening oxidative stress and inflammation [38]. In our study, we found that the significant increase in Lactobacillus content following the addition of *Bacillus coagulans* to the diet may contribute to the reduction in hepatic fat deposition in laying hens. However, since some mechanistic insights derive from mammalian models, extrapolating these microbiome–lipid interactions to the unique hepatic metabolism of laying hens requires caution and further validation.

Bile acids are crucial metabolites of gut microbes that regulate host lipid metabolism [39]. In this experiment, the inclusion of *Bacillus coagulans* significantly decreased the levels of Tα-MCA but significantly increased the levels of GCDCA, TUDCA, THDCA, LCA, and HDCA in the ileum. Notably, among the altered bile acids, GCDCA, THDCA, and TUDCA exhibited the most pronounced increases (approximately 2.9- to 3.4-fold) following *Bacillus coagulans* supplementation. Because laying hens rely almost exclusively on massive hepatic de novo lipogenesis to supply yolk precursors, efficient lipid emulsification mediated by primary bile acids like GCDCA is critical for sustaining high production. Activating intestinal *FXR* engages the gut–liver axis to curb bile acid synthesis and drive *PPARα*-mediated hepatic fatty-acid β-oxidation [40]. In a mouse model of nonalcoholic fatty liver disease (NAFLD), ligand activation of *FXR* can induce the expression of *PPARα*, promoting TG degradation and fatty acid oxidation. This synergy safeguards lipid balance and curbs hepatic fat buildup [41]. Previous studies indicate that shifts in the gut microbiome are closely associated with alterations in bile acid pools, which may subsequently be linked to intestinal *FXR* activation and the restraint of fat deposition [42]. Various endogenous bile acids can modulate *FXR* activity; among these, Tα-MCA acts as an inhibitor of *FXR*, whereas GCDCA, TUDCA, LCA, and HDCA serve as activators of *FXR* [43]. GCDCA and TUDCA curb lipid storage and inflammatory signals in the white adipose tissue of HFD-obese mice through *FXR* activation [44]. In our experiments, the decrease in Tα-MCA and the increase in GCDCA, TUDCA, LCA, and HDCA may have contributed to the upregulation of FXR and FGF19 expression in the ileum, leading to a decrease in hepatic *CYP7A1* expression, which inhibits hepatic bile acid production, thereby promoting the oxidative catabolism of fatty acids in the liver. Additionally, as most bile acids in the ileum are transported to the liver via the portal vein, the increase in GCDCA, TUDCA, LCA, and HDCA in the ileum resulting from *Bacillus coagulans* may also directly activate hepatic *FXR* through the enterohepatic cycle, further reducing hepatic fatty acid deposition in laying hens.

Oxidative stress progressively increases with aging, which often results in diminished productive performance in older laying hens. It is widely recognized that egg production performance correlates with the processes of follicular development and ovulation, influenced by reproductive hormones [45]. Ovarian aging plus oxidative-stress-driven apoptosis lowers reproductive and erodes egg output in aged hens. The current study demonstrated that the dietary inclusion of *Bacillus coagulans* significantly elevated serum reproductive levels of E_2_, P_4_, LH, and FSH while mitigating ovarian apoptosis. Additionally, prior research suggests that the reduction in apoptosis may be associated with the alleviation of oxidative stress [13]. Antioxidant enzyme activities together with MDA and H_2_O_2_ levels are routinely used to gauge systemic oxidative stress, while the redox-sensitive transcription factor Nrf2 counters this stress by boosting antioxidant enzyme activity [46]. The fermentation of polysaccharides by *Bacillus coagulans* has been reported to elevate antioxidant enzyme activities, including SOD, CAT, and GSH-Px, while decreasing MDA levels in Macrobrachium nipponense [47]. Our findings demonstrate that dietary inclusion of *Bacillus coagulans* effectively mitigates age-related reproductive decline in laying hens. The improved laying rate is primarily driven by enhanced ovarian free radical scavenging, evidenced by reduced MDA levels; increased T-SOD and CAT activities; and the significant upregulation of *Nrf2*, *HO-1*, and *SOD1* within the *Nrf2* signaling pathway. Although our qPCR results revealed significant upregulation of *Nrf2*, *HO-1*, and *SOD1* at the transcriptional level, the exact activation of these signaling cascades requires further validation at the protein level. Together, these structural and metabolic improvements likely account for the higher laying rate in aged hens.

## 5. Conclusions

In conclusion, dietary supplementation with *Bacillus coagulans* significantly improved the egg production performance of aged laying hens. This improvement is likely associated with the modulation of the gut–liver–ovary axis. Specifically, *Bacillus coagulans* reshaped the intestinal microbiota by enriching beneficial taxa and elevated the ileal levels of secondary bile acids. These metabolic shifts were accompanied by the transcriptional upregulation of genes within the *FXR/PPARα/ACOX* network, suggesting a potential promotion of hepatic fatty acid β-oxidation to alleviate steatosis. Concurrently, enhanced ovarian antioxidant capacity, reduced follicular apoptosis, and restored reproductive hormone synthesis were linked to the increased mRNA expression of *Nrf2* signaling-related genes. Overall, *Bacillus coagulans* serves as an effective nutritional strategy to combat age-related lipid metabolism disorders and ovarian decline in late-phase laying hens, though further protein-level validations are warranted to confirm the exact molecular cascades.

## Figures and Tables

**Table 1:** Effect of *Bacillus coagulans* on production performance of aged laying hens.

Content	CON	BC	SEM	*p*-Value
1–28 D				
Mean Egg Weight, g	62.88	61.66	0.288	0.284
ADFI, g/d/bird	108.67	101.64 *	1.823	0.031
Egg production, %	83.06	85.03 *	0.537	0.018
Egg mass, g/d/bird	52.13	53.23	0.507	0.300
Feed-to-egg ratio	2.04	1.95	0.038	0.142
28–56 D				
Mean Egg Weight, g	62.48	61.83	0.184	0.348
ADFI, g/d/bird	110.01	103.33 *	1.896	0.044
Egg production, %	82.73	85.19	0.912	0.086
Egg mass, g/d/bird	51.69	51.74	0.616	0.971
Feed-to-egg ratio	2.11	1.96 *	0.031	0.042
1–56 D				
Mean Egg Weight, g	62.69	61.74	0.214	0.215
ADFI, g/d/bird	109.33	102.67 *	1.944	0.038
Egg production, %	82.56	84.96 *	0.384	0.044
Egg mass, g/d/bird	52.21	52.62	0.497	0.699
Feed-to-egg ratio	2.07	1.95 *	0.030	0.049

All data are expressed as mean ± SEM, n = 6.* *p* < 0.05, CON: (basal diet); BC: (basal diet + 3 × 10^9^ CFU/kg *Bacillus coagulans*).

## Data Availability

The original contributions presented in this study are included in the article. Further inquiries can be directed to the corresponding author.

## Supplementary Materials

The following supporting information can be downloaded at https://www.mdpi.com/article/10.3390/ani16162466/s1, Table S1: Composition and nutrient levels of the basal diets; Table S2: Primer sequence of target and reference genes; Table S3: Effect of *Bacillus coagulans* on the ileal microbial community; Table S4: Effect of *Bacillus coagulans* on intestinal bile acid distribution in laying hens.

## References

[1] Shini A., Shini S., Bryden W. (2019). Fatty liver haemorrhagic syndrome occurrence in laying hens: Impact of production system. Avian Pathol..

[2] Rozenboim I., Mahato J., Cohen N., Tirosh O. (2016). Low protein and high-energy diet: A possible natural cause of fatty liver hemorrhagic syndrome in caged White Leghorn laying hens. Poult. Sci..

[3] Weiskirchen R., Lonardo A. (2025). The Ovary–Liver Axis: Molecular Science and Epidemiology. Int. J. Mol. Sci..

[4] Liu X.-t., Lin X., Mi Y.-l., Zeng W.-d., Zhang C.-q. (2018). Age-related changes of yolk precursor formation in the liver of laying hens. J. Zhejiang Univ.-Sci. B.

[5] Yang Z., Zhang J., Yuan Q., Wang X., Zeng W., Mi Y., Zhang C. (2024). Flavonoid Fisetin Alleviates Ovarian Aging of Laying Chickens by Enhancing Antioxidant Capacity and Glucose Metabolic Homeostasis. Antioxidants.

[6] Mazhar S., Simon A., Khokhlova E., Colom J., Leeuwendaal N., Deaton J., Rea K. (2024). In vitro safety and functional characterization of the novel Bacillus coagulans strain CGI314. Front. Microbiol..

[7] Zhao Z., Sun M., Cui X., Chen J., Liu C., Zhang X. (2023). Bacillus coagulans MZY531 alleviates intestinal mucosal injury in immunosuppressive mice via modulating intestinal barrier, inflammatory response, and gut microbiota. Sci. Rep..

[8] Hakami Z.M., Alhotan R.A., Al Sulaiman A.R., Aljumaah R.S., Palombo V., D’Andrea M., Alharthi A.S., Abudabos A.E. (2025). Enhancing Laying Hen Productivity and Health: Influence of Dietary Probiotic Bacillus Strains and Prebiotic Saccharomyces cerevisiae Yeast Cell Wall on Production Performance, Egg Quality, and Inflammatory Responses. Animals.

[9] Wang X., Jian H., Zhao W., Li J., Zou X., Dong X. (2023). Effects of dietary Bacillus coagulans on the productive performance, egg quality, serum parameters, and intestinal morphology of laying hens during the late laying period. Ital. J. Anim. Sci..

[10] Almalamh N.A., Aljumaah R.S., Alharthi A.S., Al-Garadi M.A., Albaadani H.H., Hakami Z.M., Abudabos A.E. (2024). Effects of three different strains of Bacillus-based probiotics and Saccharomyces cerevisiae-based prebiotic on performance, egg components and gene expression of laying hens during phase II of production. Ital. J. Anim. Sci..

[11] Abudabos A.E., Hakami Z.M., Al Sulaiman A.R., Aljumaah R.S., Palombo V., Aljumaah M.R., D’Andrea M., Alharthi A.S., Alhotan R.A. (2025). RNA Sequencing-Based Transcriptome Analysis of Liver in Laying Hens Supplemented with Dietary Probiotic Bacillus Species and Prebiotic Yeast (*Saccharomyces cerevisiae*) Cell Walls. Vet. Sci..

[12] Amevor F.K., Cui Z., Du X., Ning Z., Shu G., Jin N., Deng X., Tian Y., Zhang Z., Kang X. (2021). Combination of Quercetin and Vitamin E Supplementation Promotes Yolk Precursor Synthesis and Follicle Development in Aging Breeder Hens via Liver–Blood–Ovary Signal Axis. Animals.

[13] Yang X., Zheng H., Niu J., Chen X., Li H., Rao Z., Guo Y., Zhang W., Wang Z. (2024). Curcumin alleviates zearalenone-induced liver injury in mice by scavenging reactive oxygen species and inhibiting mitochondrial apoptosis pathway. Ecotoxicol. Environ. Saf..

[14] Huang Z., Liu H., Wang G., Ge H., Shi Y., Feng J., Li C., Zhang M. (2025). Dietary glycyrrhizin enhances reproductive performance by improving intestinal microbiota, liver lipid metabolism and ovarian senescence in aged breeder hens. J. Anim. Sci. Biotechnol..

[15] Guo Y., Wang B., Luo Q. (2026). Rehmanniae Radix Powder Enhances Antioxidative Capacity, Immunity, and Gut Health in Late-Phase Laying Hens. Animals.

[16] Arulnathan V., Turner I., Bamber N., Ferdous J., Grassauer F., Doyon M., Pelletier N. (2024). A systematic review of potential productivity, egg quality, and animal welfare implications of extended lay cycles in commercial laying hens in Canada. Poult. Sci..

[17] Kim H.-J., Kim H., Yu M., Lim C., Hong J., Hong E.-C., Kim Y. (2026). Effects of Dietary Choline Supplementation on Production Performance and Liver Characteristics in Late-Laying Hens. Animals.

[18] Li H., Hou Y., Hu J., Li J., Liang Y., Lu Y., Liu X. (2023). Dietary naringin supplementation on hepatic yolk precursors formation and antioxidant capacity of Three-Yellow breeder hens during the late laying period. Poult. Sci..

[19] Zhang J., Zhang J., Li K., Fu X., Liang Y., Zhang M., Zhuang S., Gao Y. (2024). Kaempferol and Vitamin E Improve Production Performance by Linking the Gut-Uterus Axis Through the Reproductive Hormones and Microbiota of Late-Laying Hens. Animals.

[20] Tsai M.Y., Shih B.L., Liaw R.B., Chen W.T., Lee T.Y., Hung H.W., Hung K.H., Lin Y.F. (2023). Effect of dietary supplementation of Bacillus subtilis TLRI 211-1 on laying performance, egg quality and blood characteristics of Leghorn layers. Anim. Biosci..

[21] Wu T., Wang G., Xiong Z., Xia Y., Song X., Zhang H., Wu Y., Ai L. (2022). Probiotics Interact With Lipids Metabolism and Affect Gut Health. Front. Nutr..

[22] Liu C., Radebe S.M., Zhang H., Jia J., Xie S., Shi M., Yu Q. (2022). Effect of Bacillus coagulans on maintaining the integrity intestinal mucosal barrier in broilers. Vet. Microbiol..

[23] Li H., Wang T., Xu C., Wang D., Ren J., Li Y., Tian Y., Wang Y., Jiao Y., Kang X. (2015). Transcriptome profile of liver at different physiological stages reveals potential mode for lipid metabolism in laying hens. BMC Genom..

[24] Zaefarian F., Abdollahi M.R., Cowieson A., Ravindran V. (2019). Avian Liver: The Forgotten Organ. Animals.

[25] Xuan Y., Wu D., Zhang Q., Yu Z., Yu J., Zhou D. (2024). Elevated ALT/AST ratio as a marker for NAFLD risk and severity: Insights from a cross-sectional analysis in the United States. Front. Endocrinol..

[26] Amevor F.K., Uyanga V.A., Wu L., Xu D., Shu G., Wang Y., Zhao X. (2025). Enhancing poultry health and productivity through the liver-gut axis with integrated nutritional and immunological approaches: A mini-review. Front. Physiol..

[27] Wang Y., Zhang C., Chen X., Zheng A., Liu G., Ren Y., Chen Z. (2024). Dietary supplementation of compound probiotics to improve performance, egg quality, biochemical parameters and intestinal morphology of laying hens. Front. Vet. Sci..

[28] Lu L., Wang L., Yang M., Wang H. (2025). Role of METTL16 in PPARγ methylation and osteogenic differentiation. Cell Death Dis..

[29] Guilherme A., Rowland L.A., Wetoska N., Tsagkaraki E., Santos K.B., Bedard A.H., Henriques F., Kelly M., Munroe S., Pedersen D.J. (2023). Acetyl-CoA carboxylase 1 is a suppressor of the adipocyte thermogenic program. Cell Rep..

[30] Shi B., Chen J., Guo H., Shi X., Tai Q., Chen G., Yao H., Mi X., Zhong R., Lu Y. (2025). ACOX1 activates autophagy via the ROS/mTOR pathway to suppress proliferation and migration of colorectal cancer. Sci. Rep..

[31] Li Y., Wang L., Yi Q., Luo L., Xiong Y. (2024). Regulation of bile acids and their receptor FXR in metabolic diseases. Front. Nutr..

[32] Tschuck J., Theilacker L., Rothenaigner I., Weiß S.A.I., Akdogan B., Lam V.T., Müller C., Graf R., Brandner S., Pütz C. (2023). Farnesoid X receptor activation by bile acids suppresses lipid peroxidation and ferroptosis. Nat. Commun..

[33] Mori H., Svegliati Baroni G., Marzioni M., Di Nicola F., Santori P., Maroni L., Abenavoli L., Scarpellini E. (2022). Farnesoid X Receptor, Bile Acid Metabolism, and Gut Microbiota. Metabolites.

[34] Wang Y., Wu J., Lv M., Shao Z., Hungwe M., Wang J., Bai X., Xie J., Wang Y., Geng W. (2021). Metabolism Characteristics of Lactic Acid Bacteria and the Expanding Applications in Food Industry. Front. Bioeng. Biotechnol..

[35] Hua M., Wang J., Li Y., He Y., Luo Z., Li D., Sun M., Miao X., Niu H., Pan T. (2026). Gut-Centric Multi-System Regulation by Bacillus subtilis and Bacillus natto: A Review of Their Probiotic Functions in Nutrition, Immunity, and Metabolism. Nutrients.

[36] Zhao H., Luo Y.e., Zhang Y., Chen X., Wang H., Guo D., Wu Z. (2020). Effects of Bacillus subtilis on hepatic lipid metabolism and oxidative stress response in grass carp (*Ctenopharyngodon idellus*) fed a high-fat diet. Mar. Life Sci. Technol..

[37] Yoon Y., Kim G., Noh M.G., Park J.H., Jang M., Fang S., Park H. (2020). Lactobacillus fermentum promotes adipose tissue oxidative phosphorylation to protect against diet-induced obesity. Exp. Mol. Med..

[38] Abdi M., Esmaeili Gouvarchin Ghaleh H., Ranjbar R. (2022). Lactobacilli and Bifidobacterium as anti-atherosclerotic agents. Iran. J. Basic. Med. Sci..

[39] Staley C., Weingarden A.R., Khoruts A., Sadowsky M.J. (2017). Interaction of gut microbiota with bile acid metabolism and its influence on disease states. Appl. Microbiol. Biotechnol..

[40] Chiang J.Y., Pathak P., Liu H., Donepudi A., Ferrell J., Boehme S. (2017). Intestinal Farnesoid X Receptor and Takeda G Protein Couple Receptor 5 Signaling in Metabolic Regulation. Dig. Dis..

[41] Fang T., Wang H., Pan X., Little P.J., Xu S., Weng J. (2022). Mouse models of nonalcoholic fatty liver disease (NAFLD): Pathomechanisms and pharmacotherapies. Int. J. Biol. Sci..

[42] Larabi A.B., Masson H.L.P., Bäumler A.J. (2023). Bile acids as modulators of gut microbiota composition and function. Gut Microbes.

[43] Chiang J.Y.L., Ferrell J.M. (2022). Discovery of farnesoid X receptor and its role in bile acid metabolism. Mol. Cell Endocrinol..

[44] Freitas I.N., da Silva J.A., de Oliveira K.M., Lourençoni Alves B., Dos Reis Araújo T., Camporez J.P., Carneiro E.M., Davel A.P. (2023). Insights by which TUDCA is a potential therapy against adiposity. Front. Endocrinol..

[45] Agarwal A., Gupta S., Sharma R.K. (2005). Role of oxidative stress in female reproduction. Reprod. Biol. Endocrinol..

[46] Guo S., Li H., Peng B., Lv Z., Duan Y., Niu J., Guo Y., Wang Z., Zhang W. (2026). Repairing effect of total glucosides of Paeonia on deoxynivalenol-induced enterohepatic axis damage in weaned piglets. Ecotoxicol. Environ. Saf..

[47] Wang Y., Liang Y., Yu J., Li Z., Wang W., Jiang L., Liu B. (2024). Effects of polysaccharide fermentation with Bacillus coagulans on growth, antioxidant and immunity of Macrobrachium nipponense (riental river prawn). Front. Mar. Sci..

