# Supplementary material for: Bacillus coagulans Improves Performance by Modulating Bile Acid Metabolism to Suppress Hepatic Lipid Accumulation and Attenuating Ovarian Oxidative Stress in Late Laying Hens
Source: Animals (Basel). 2026 Aug 8;16(16):2466. doi: 10.3390/ani16162466 (PMC13508888; doi:10.3390/ani16162466)
Supplement: Supplementary file 1 [file animals-16-02466-s001.zip › animals-4465435-supplementary.pdf]

Table S1 Composition and nutrient levels of the basal diets

Ingredient	Content %	Nutrient level [2]	Content
Corn (86% DM)	63.8	Metabolizable energy,Mcal/kg	2.73
Soybean meal (46% CP)	16.2	Crude protein, %	16.12
Corn protein powder (60% CP)	3.0	Lysine, %	0.82
Wheat Bran	4.0	Methionine, %	0.53
Limestone	9.0	Cysteine+methionine, %	0.71
Dicalcium phosphate	1.0	Calcium, %	3.65
Soybean oil	0.5	Available phosphorus, %	0.41
NaCl	0.3		
Methionine	0.2		
Premix [1]	2.0		
Total	100.0		

[1]The premix provided the following per kg of the diet: vitamin A, 12,500 IU; vitamin D3, 4,000 IU; vitamin K3, 2 mg; thiamine, 1 mg; riboflavin, 8.5 mg; calcium pantothenate, 50 mg; niacin acid, 32.5 mg; pyridoxine, 8 mg; folic acid, 5 mg; B12, 5 mg; choline chloride, 500 mg; iron, 60 mg; copper, 10 mg; manganese, 80 mg; zinc 80 mg; iodine 0.3 mg; and selenium 0.30 mg.

[2]Metabolizable energy and available phosphorus in the nutrient levels were calculated values, while the others were measured values.

Note: For the BC group diet, the commercial *Bacillus coagulans* product (1×10^{10} CFU/g) was additionally supplemented to the basal diet at a dosage of 0.3 g/kg to achieve a final concentration of 3×10^{9}CFU/kg.

TableS2.Primer sequence of target and reference genes.

Gene[1]	GenBank number	primer (5'-3')[2]	Product size(bp)
FXR	NM_001396910.1	F: TCAGGAGCCGCTGCTGGATATC<br>R: CCCAGGAGGCACGCAAAGTG	84
ACC	XM_046929954.1	F: CAGACGGTTGTGGTTGGCAGAG<br>R: TGGCGGGATCAGCAGGGATG	103
FAS	NM_205155.4	F: AGCCATTGCCATCGCCTTGAG<br>R: GCTGTGGGAACCTTGCTTGGAG	95
SREBP-1	NM_204126.3	F: AGGCGGAGGTGATGGAGATGC<br>R: CTCGGAGTCACTGCTGCTGTTG	113
LXRα	NM_204542.3	F: GAGGTGATGTTGTTGGAGACA<br>R: GGCGAAGTCATCCCGATTATAG	96
ACOX1	XM_015295163.4	F: CAGGTGAACCAGCGTGTGAAGG<br>R: CCGGCCAAGCACAGATCCAAG	111
CYP7A1	NM_001001753.2	F: ACCCAGCAGAAGGAAACACCAC<br>R: TGAGGGCATCTAGGGCGTTACC	81
PPARα	XM_046942569.1	F: CATGGCAGAGAAGACGCTGGTG<br>R: ACTGGCAGCAGTGGAAGATTCG	89
PPARγ	NM_001001460.2	F: GCCACAAGCGGAGAAGGAGAAG<br>R: CCCTTGCCTTGGCTTTGGTCAG	149
FGF19	NM_204674.3	F: CAGCAAGCACGGGCTCTTCAG<br>R: ACTCTGCGGGCTCTGGCTAC	85
APOB	NM_001044633.2	F: GGTCTAGCCAGCAGTGCAACTAC<br>R: ACAGTGACACAGAGCCTCCAGAG	103
APOV1	NM_205483.3	F: ACGAAGCTGTGAACAAGGTGTCC<br>R: CCGCCAGTTTAGTGAGTCTAGCC	126
VTG2	NM_001031276.2	F: TGCCTCCTCAACTGCCACCTC<br>R: CTCCTGCTGCTGCTGCTTGC	132
ESR1	NM_205183.2	F: AGGAACGGGGAGGCTTCTTCTAC<br>R: CTTCCAGCAAGGCACTGACCATC	136
ESR2	NM_001396358.1	F: GGCTCAAGAAGAGAACGCTGTGG<br>R: TCTGGACTGACTGTGCTGAGGAG	137
FSHR	NM_205079.2	F: ACCTATGCCATGCAACTCAACCG<br>R: GGGAAGAAGTGCCACCGTGAAAG	99
LHR	NM_204936.2	F: CACGGTTCCTCGCTTCCTCATG<br>R: CCGCTGCCTGTTTGCCAGTC	141
Nrf2	NM_001396902.1	F: GGGACGGTGACACAGGAACAAC<br>R: GCTCTCCACAGCGGGAAATCAG	97
HO-1	NM_205344.2	F: GCTGGGAAGGAGAGTGAGAGGAC<br>R: GCGACTGTGGTGGCGATGAAG	107
NQO1	NM_001277619.2	F: CACTTCTGCGGCTTCCAGGTG	104

		R: AGGCGGCTCTTCCATGTACTCAG	
SOD1	NM_205064.2	F: GGTGACCTCGGCAATGTGACTG<br>R: GATGCAGTGTGGTCCGGTAAGAG	90
GPX1	NM_001277853.3	F: AAGTGCTGCTGGTGGTCAACG<br>R: GTTCTCCTGGTGCCCGAATTGG	146
β-Actin	NM_205518.2	F: TGGATCTGGCTGGCCGTGAC<br>R: TCTCTCGGCTGTGGTGGTGAAG	86

[1]*FXR*=Farnesoid X Receptor; *ACC*= Acetyl-CoA carboxylase; *FAS*= Fatty acid synthase; *SREBP-1*= Sterol regulatory element-binding protein-1; *LXRα*= liver X-activated receptor α; *ACOX1*= Peroxisomal acyl-coenzyme A oxidase 1; *CYP7A1*= Cholesterol 7-alpha hydroxylase; *PPARα*= Perixisome proliferation-activated receptor alpha； *PPARγ*= Perixisome proliferation-activated receptor gamma; *FGF19*= Fibroblast growth factor 19; *APOB*= Apolipoprotein B; *APOV1*= Apovitellenin 1；*VTG2*= Vitellogenin 2; *ESR1*= Estrogen receptor 1; *ESR2*= Estrogen receptor 2; *FSHR*= Follicle stimulating hormone receptor; *LHR*= Luteinizing hormone receptor；*Nrf2*= Nuclear factor erythroid 2-related factor 2； *HO-1*= Heme oxygenase 1; *NQO1*= NAD(P)H quinone dehydrogenase 1; *SOD1*= Superoxide dismutase 1; *GPX1*= Glutathione peroxidase 1; *β-Actin*= Beta-actin

[2] F, Forward; R, Reverse.

Table S3 Effect of *Bacillus coagulans* on the ileal microbial community

Content	CON	BC	SEM	*P*-value
**Phyla**				
Firmicutes	94.46	97.71**	0.864	0.008
Actinobacteriota	3.63	1.97*	0.609	0.036
**Classes**				
Bacilli	91.00	97.17***	0.505	0.001
Actinobacteria	3.36	1.91***	0.232	0.001
Clostridia	0.32	0.28	0.035	0.247
**Orders**				
Lactobacillales	85.51	86.21	1.656	0.682
Bacillales	3.05	6.70***	0.249	0.001
Micrococcales	1.78	0.66***	0.255	0.001
Bifidobacteriales	0.14	0.49*	0.132	0.024
Corynebacteriales	0.16	0.26	0.048	0.056
Lachnospirales	0.15	0.05*	0.035	0.034
Peptostreptococcales	0.25	0.27	0.204	0.947
**Families**				
Lactobacillaceae	82.58	85.27***	0.531	0.001
Bacillaceae	3.05	6.70**	0.249	0.012
Micrococcaceae	2.12	3.19**	0.124	0.012
Enterococcaceae	0.45	0.48	0.057	0.686
Bifidobacteriaceae	0.14	0.46***	0.021	＜0.001
Streptococcaceae	0.31	0.13***	0.015	＜0.001
Corynebacteriaceae	0.16	0.26	0.047	0.057
**Genera**				
Lactobacillus	82.58	85.27***	0.531	＜0.001
Bacillus	3.05	6.62***	0.703	0.001
Rothia	1.72	0.62*	0.248	0.022
Enterococcus	0.48	0.46	0.141	0.848
Aeriscardovia	0.18	0.47	0.147	0.080
Streptococcus	0.32	0.13**	0.048	0.003
Corynebacterium	0.17	0.22	0.074	0.490

All data are expressed as mean ± SEM, n=5. ns $P>0.05$, no significant difference; * $P<0.05$, ** $P<0.01$, *** $P<0.001$. CON: (basal diet); BC: (basal diet＋3×10^9 CFU/kg *Bacillus coagulans*)

Table S4 Effect of *Bacillus coagulans* on intestinal bile acid distribution in laying hens

Content	CON	BC	SEM	*P*-value
**Primary bile acids**				
Tα-MCA	30882.83	11423.79***	1743.93	0.001
Tβ-MCA	423.33	450.09	149.51	0.862
GCA	301.16	1328.61	591.44	0.113
α-MCA	1136.86	1587.52	566.12	0.44
β-MCA	469.39	904.04	239.83	0.108
CDCA	701077.60	770931.35	145844.12	0.642
CA	89577.46	95376.62	53049.42	0.915
GCDCA	1690.85	5829.43**	986.10	0.004
TCDCA	375426.02	447405.76	385942.13	0.856
**Secondary bile acids**				
TUDCA	2778.92	8078.21***	641.72	0.001
THDCA	3323.78	10119.33**	2176.67	0.011
UDCA	1873.33	7124.74	3024.38	0.113
7-KDCA	984.58	774.22	500.79	0.683
12-DHCA	828.70	684.38	421.96	0.739
DHCA	44.77	42.92	9.53	0.850
LCA	973.00	1556.81**	127.14	0.005
7-KLCA	4797.59	6761.35	887.75	0.051
12-KLCA	407.90	1015.62	310.86	0.079
DCA	320.96	311.96	250.10	0.972
ApCA	66.70	36.79	18.64	0.164
ACA	1735.85	3064.76	1248.40	0.312
HDCA	3011.51	4968.57**	682.60	0.017
TLCA	441.83	510.23	279.78	0.812

All data are expressed as mean ± SEM, n=6. ns *P*>0.05, no significant difference; ** *P*<0.01, *** *P*<0.001. CON: (basal diet); BC: (basal diet＋3×10^9 CFU/kg *Bacillus coagulans*)
